# Supplementary material for: Differences in workplace violence and health variables among professionals in a hospital emergency department: A descriptive-comparative study
Source: PLoS One. 2024 Dec 5;19(12):e0314932. doi: 10.1371/journal.pone.0314932 (PMC11620588; doi:10.1371/journal.pone.0314932)
Supplement: S1 File — (DOCX) [file pone.0314932.s001.docx]

**SUPPLEMENTARY FILE 1: ITEMS BELONGING TO EACH VARIABLE**

Scores are obtained from the mean of the set of items belonging to each variable. Specifically:

- Non-physical user violence (mean of items 18-24).
- Physical user violence (mean of items 25-27).
- Lateral personal violence (mean of items 28, 29, 30, 33).
- Lateral relational violence (mean of items 31, 32, 34).
- Lateral work-related violence (mean of items 35-37).
- Depression (mean of items 66-72)
- Anxiety (mean of items 52-58)
- Somatic symptoms (mean of items 45-51)
- Social dysfunction (mean of items 59-65)
- Emotional exhaustion (mean of items 73, 74, 75, 76 and 78)
- Professional efficacy (mean of items 77, 79, 82, 83, 84 and 87)
- Cynicism (mean of items 84 and 87), 84 and 87)
- Cynicism (mean of items 80, 81, 85, 86)
- Vigor (mean of items 88, 89, 92)
- Dedication (mean of items 90, 91, 94)
- Absorption (mean of items 93, 95, 96)
- Intrinsic job satisfaction (mean of items 193, 194, 195, 196, 197, 200, 202, 204, 205, 206, 208, 212).
- Extrinsic Job Satisfaction (mean of items 198, 199, 201, 203, 210, 211)
- Overall Job Satisfaction (mean of items 207, 209)
