# Supplementary material for: Differences in workplace violence and health variables among professionals in a hospital emergency department: A descriptive-comparative study
Source: PLoS One. 2024 Dec 5;19(12):e0314932. doi: 10.1371/journal.pone.0314932 (PMC11620588; doi:10.1371/journal.pone.0314932)
Supplement: Attachment — Submitted filename: Response to Reviewers (1).docx [file pone.0314932.s003.docx]

Dear Editor,

We are very grateful to you for considering our article for possible publication in the Plos One Journal.

We have received and studied the reviewers' comments. Thanks to your input, the study has been significantly improved. At the end of this letter, we present the comments received and how they have been addressed in the article.

Regards.

Paloma López Ros.

We then proceed to answer the reviewers' questions.

**REVIEWER 2**

**There are still problems with the data presented in table 1 (to be revised again by the authors).**

We are grateful for your comment. We are sorry for the mistakes in this regard. The relevant errors have been rechecked and amended. Again, we thank you for your observations that have improved our study.

**In page 4 the authors state that the study population consists of 120 professionals, but it seems that this number is the final number of professionals that answered the questionarires (so, the size of the final sample).**

Thanks for your comment. It has been specified that, indeed, the final sample size is 120.

**There continues to be problems in the way the authors describe the sample selection (at least they should specify the total size of the population, the selection method, the response rate, and some comments and data on the differences between the initial sample and the final sample).**

Thanks for your comment. This information is described in detail in the Procedure section

**There continues to be some problems with the categorization of the professionals. And some curious result: It seems that physicians show higher levels of satisfaction than medical staff (which is the differene between physicians and medical staff?): this should be adressed ensuring that the names of the different categories of study are the same through all the manuscript, including the tables..**

Thanks for your feedback. Your comments highly improve our study. We have made sure that the different categories have the same name throughout the study so that there is no confusion.

**Regarding the gender issue: as the authors have data on the gender of the professionals, if they think that gender is a relevant issue, they should present the data and conclussions of this study relative to gender. The discussion section must be generated by the comparison of the data and conclussions of the study and other relevant comparable studies in the bibliography.**

**The same applies to the discussion on job satisfaction, burnout or engagement: The authors make comments on different aspects that influence these variables, but they do not compare with the data on their study, making the discussion less meaningful..**

We greatly appreciate your comment. As you recommend, we believe that the discussion includes a comparison of our results and conclusions with those of previous literature. This is reflected, for example, in the second and third paragraphs, where we specifically comment on the differences found between professionals. In the sixth, seventh and eighth paragraphs, you can also find a comparison with previous evidence. This covers the main objective of our study, analyzing the differences between the different professional categories.

It is a very interesting proposal, analyzing gender and other variables in relation to workplace violence, satisfaction, burnout, engagement... However, this differs from the objective of our study and would unfortunately exceed the length limit of the manuscript.

In our discussion we have considered covering a broader view that contextualizes the reader on all the influential variables according to the topic, going beyond the results obtained in the study. We consider that this is an interesting debate to discuss, in line with future research proposals in relation to our study, as our main objective. Once again, thank you very much for your comment.
