# Supplementary material for: Differences in workplace violence and health variables among professionals in a hospital emergency department: A descriptive-comparative study
Source: PLoS One. 2024 Dec 5;19(12):e0314932. doi: 10.1371/journal.pone.0314932 (PMC11620588; doi:10.1371/journal.pone.0314932)
Supplement: Attachment — Submitted filename: Response to Reviewers (2).docx [file pone.0314932.s004.docx]

Dear Editor,

We are very grateful to you for considering our article for possible publication in the Plos One Journal.

We have received and studied the reviewers' comments. Thanks to your input, the study has been significantly improved. At the end of this letter, we present the comments received and how they have been addressed in the article.

Regards.

Paloma López Ros.

We then proceed to answer the reviewers' questions.

**REVIEWER 2**

**This paragraph in the Results section of the Summary should be revised as it is incongruent (physicians cannot show hiogher levels of satisfaction than medical staff as they are the same group):
"Regarding job satisfaction, nurses report higher intrinsic satisfaction, while physicians, nurses and auxiliary nursing staff show higher levels of extrinsic satisfaction compared to administrative and medical staff."**

We are grateful for your comment. We are sorry for the mistakes in this regard. The relevant errors have been rechecked and amended.

“Regarding job satisfaction, nurses report higher intrinsic satisfaction. Medical staff, nurses and nursing assistants show higher levels of extrinsic satisfaction compared to administrative staff”

**In the same direction, this paragraph in the Results section of the text should be revised (lines 324-326) as it is incongruent (medical staff cannot at the same time show greater and lower extrinsic satisfaction thanm administration group):
"In extrinsic satisfaction, medical staff, nursing staff and nursing assistants showed significantly higher scores than the administration group and this, in turn, than the medical staff"**

Thank you for your comment. We have clarified this section and corrected it in the manuscript.

“In extrinsic satisfaction, medical staff, nursing staff and nursing assistants showed significantly higher scores than the administration group (χ²= 30.21; p< .001). In this case, the magnitude of the effect was high “
